# Impact of contralateral prophylactic mastectomy on survival outcomes in patients with unilateral breast cancer: A systematic review and meta-analysis

**DOI:** 10.12669/pjms.40.8.9708

**Published:** 2024-09

**Authors:** Min Yao, Puchao Peng, Lijie Chen, Zhouming Xu

**Affiliations:** 1 Min Yao, Department of Breast Surgery, Huzhou Maternity & Child Health Care Hospital, Huzhou, Zhejiang Province 313000, P.R. China; 2 Puchao Peng, Department of Breast Surgery, Huzhou Maternity & Child Health Care Hospital, Huzhou, Zhejiang Province 313000, P.R. China; 3 Lijie Chen, Department of Breast Surgery, Huzhou Maternity & Child Health Care Hospital, Huzhou, Zhejiang Province 313000, P.R. China; 4 Zhouming Xu, Department of Breast Surgery, Huzhou Maternity & Child Health Care Hospital, Huzhou, Zhejiang Province 313000, P.R. China

**Keywords:** Contralateral prophylactic mastectomy, Unilateral breast cancer, Overall survival, Breast cancer-specific survival, Recurrence free survival, Contralateral breast cancer, Systematic review, Meta-analysis

## Abstract

**Objective:**

To synthesize contemporary evidence of the impact of contralateral prophylactic mastectomy (CPM) on survival outcomes in patients with unilateral breast cancer (UBC).

**Methods:**

PubMed, EMBASE and Scopus databases were searched for observational studies published up to November 15, 2023. Random-effects model was used to obtain pooled effect estimates that were reported as hazards ratio (HR) with 95% confidence intervals (CI). The outcomes of interest were overall survival (OS), breast cancer-specific survival (BCSS), recurrence free survival (RFS) and risk of contralateral breast cancer (CBC).

**Results:**

Twenty-one studies were included. Most studies had a retrospective design. CPM was associated with significant improvement of OS (HR 0.80, 95% CI: 0.75, 0.85), BCCS (HR 0.82, 95% CI: 0.74, 0.90), and RFS (HR 0.72, 95% CI: 0.60, 0.86) and significantly reduced risk of CBC (HR 0.05, 95% CI: 0.03, 0.09) in patients with UBC. No evidence of publication bias was detected.

**Conclusion:**

Our results provide strong evidence supporting the positive impact of CPM on survival outcomes in patients with UBC. Further research and long-term follow-up studies are warranted to validate these findings.

## INTRODUCTION

Breast cancer is the most prevalent female cancer, with about 2.3 million new cases reported worldwide every year.^1^ Despite current trends towards less invasive therapeutic approaches, rates of contralateral prophylactic mastectomy (CPM) in cases of unilateral breast cancer (UBC) more than doubled in the recent years.^2^-^4^ CPM involves the surgical removal of the healthy breast in addition to the affected breast, even in the absence of contralateral disease. The observed rise in CPM rates underscores a complex interplay of factors that impact decision-making in patients with UBC, from the wider access to personalized genetic information to advancements in post-mastectomy reconstruction options and the perceived improvement in overall survival (OS) after CPM.^5^,^6^

However, the evidence of the positive impact of CPM on the OS of patients with UBC is still controversial. Previous meta-analyses, conducted by Jia et al. and Fayanju et al., were constrained by a limited dataset and lack of contemporary studies.^7^,^8^ The review by Jia et al. included five studies with 1700 UBC patients and found that CPM correlated with a lower risk of contralateral breast cancer (CBC), and significantly increased OS and breast-cancer specific survival (BCSS).^7^ A study by Fayanju et al. included 14 reports, many predating the year 2000 and documented that CPM was linked to a significant increase in the OS of recipients when compared to non-recipients.^8^ CPM was also found to be associated with lower risks of breast cancer specific mortality and risk of CBC. With the publication of more contemporary data, there is a pressing need for an updated and comprehensive review of the recent studies. Understanding the impact of CPM on survival outcomes is crucial, as it directly informs treatment decisions and influences the evolving landscape of breast cancer care. The current review aimed to fill the existing gap by conducting a thorough literature search and synthesizing data from relevant contemporary studies to critically assess whether CPM confers a survival benefit in cases of UBC.

## METHODS

### Databases searched and search strategy

An extensive and systematic search was done in PubMed, Embase, and Scopus databases. We used a combination of keywords, and the search was confined to studies published up to November 15, 2023. Manual search of reference lists and relevant review articles was done as well.

### Inclusion and exclusion criteria

Studies of female patients with UBC, specifically examining the correlation between CPM in the apparently healthy breast, and reporting various outcomes such as OS, BCSS, recurrence-free survival (RFS), and the risk of CBC were included. The selected studies were required to include control subjects under surveillance without any surgical or non-surgical intervention in the contralateral breast. English-language, peer-reviewed observational studies (cohort studies and case-control studies), and randomized controlled trials were eligible for inclusion. To ensure the reliability of reported effect sizes in the included studies, we considered only those studies that provided adjusted effect sizes for the specified outcomes. This was done to mitigate bias in the reported associations.

We excluded studies published before the year 2000, studies where the control group underwent any form of surgical intervention or radiotherapy in the contralateral breast, and studies reporting unadjusted effect sizes. Case reports, letters, reviews, and conference abstracts were also excluded.

### Study screening and final selection

After obtaining the initial set of studies from the search across the database’s, duplicated studies were removed. Two researchers from our team independently conducted a review of the remaining studies. In the initial screening phase, titles and abstracts of each study were assessed to determine their potential relevance to the research question. Studies meeting predefined criteria were selected for further evaluation. In the subsequent stage, a detailed assessment of the full texts of the selected studies was carried out to determine their eligibility for inclusion. Any discrepancies or disagreements regarding study inclusion were resolved through discussions among the study authors. The meta-analysis adhered to the PRISMA guidelines.^9^ We registered the study protocol in PROSPERO, a prospective register for systematic reviews (Registration number [CRD42023484810]).

### Statistical analysis, including data extraction and quality assessment

Data from the final set of studies were extracted independently by two authors, using a standardized data extraction Form. In cases of any disparities, discussions were done to reach a consensus. The Newcastle-Ottawa Scale (NOS) assessed potential bias in the included studies.^10^ Pooled effect sizes were reported as hazard ratios (HR) with 95% confidence intervals (CI). A random-effects model was used for all analyses to account for the variations in the baseline characteristics. Publication bias was assessed by funnel plots and Egger’s test.^11^ P< 0.05 was statistically significant.

## RESULTS

**Fig.1:**
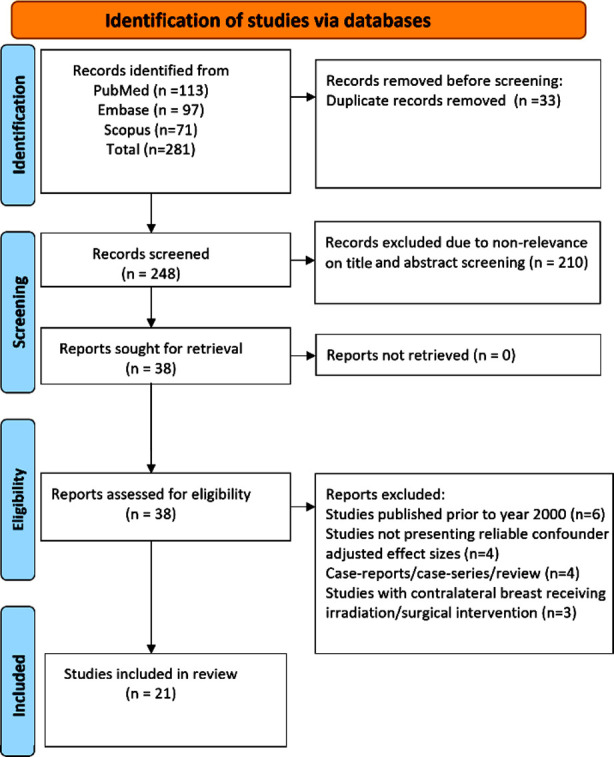
Selection process of studies included in the review.

Search of the databases identified 281 studies (Fig.1). After eliminating 33 duplicate papers, 248 unique studies underwent an initial screening based on titles and abstracts, leading to the exclusion of 210 studies, not meeting the predetermined criteria. Full texts examination of the remaining 38 studies led to the exclusion of an additional 17 studies. At the end, 21 studies were incorporated in our meta-analysis.^12^-^32^

Supplementary Table-I provides details of the included studies. All, except one, were retrospective cohort studies. Most were conducted in the United States (n=12), followed by China (n=4) and Netherlands (n=2). One study each was done in Canada, United Kingdom, and Australia (Supplementary Table-I). The included studies reported data of a total of 10,47,497 patients of them, 9,32,779 underwent unilateral mastectomy only, and 1,14,718 underwent additional contralateral mastectomy. A total of 19 studies, out of 21, reported on the stage of tumour and all of them had patients with stage one or two tumour.

**Supplementary Table-I:** Characteristics of the studies included in the meta-analysis.

Author (year of publication)	Study design	Country	Subject and tumour characteristics	Sample size	Newcastle Ottawa quality score
Wu (2023)	Retrospective cohort	China	Majority aged <65 years (81%) TNM I/II (85%) Grade 3 / 4 (72%) Triple negative (70%)	238 (119 with only unilateral mastectomy (UM) and 119 with contralateral prophylactic mastectomy (CPM))	8
Huang (2023)	Retrospective cohort	China	Majority aged >35-40 years (60%); median age of 35.5 years TNM I/II (73%) Grade 3 / 4 (60%) HR+ (67%) HER2 negative (majority; 35%) Follow up period of >5 years	26,178 (13089 with only UM and 13089 with CPM)	8
Fasano (2023)	Retrospective cohort	USA	Median age of around 50 years TNM I/II (>70%) Grade 3 / 4 (75%) Triple negative (100%) Mean follow up of 4.5 years	796 (673 with UM only and 123 with CPM)	7
Makhnoon (2022)	Retrospective cohort	USA	Median age of 40 years TNM I/II (80%) HER 2 negative (80%) HR+ (50%; majority) Median follow up of 7.9 years	144 (81 with UM only and 63 with CPM)	7
Yang (2021)	Retrospective cohort	China	Majority aged 40-59 years (72%) TNM I/II (majority; 75%) Grade 3 / 4 (83%) All Triple negative Median follow up of 35 months	6078(3039 with UM only and 3039 with CPM)	8
Chen (2019)	Retrospective cohort	China	All with age under 40 years TNM I/II (100%) HR+ (Majority; 70%) Median follow up of 113 months	4380 (2326 with UM only and 2054 with CPM)	7
Lazow (2019)	Retrospective cohort	USA	Mean age of 35 years TNM I (100%) HER 2 negative (Majority; 45%) HR+ (Majority; 45%) Mean follow up of 62 months	6785 (2646 with UM only and 4139 with CPM)	8
Wong (2017)	Retrospective cohort	USA	Majority aged more than 45 years TNM I/II (75%) HER 2 negative (Majority; 80%) HR+ (Majority; 75%) Mean follow up of 8.3 years	200628 (165888 with UM only and 34740 with CPM)	8
Heemskerk-Gerritsen (2015)	Retrospective cohort	Netherlands	Median age of around 43 years TNM I/II (Majority; >50%) Grade 3 (75%) HR+ (70%) HER 2- (85%) Median follow up of 9.5 years	583 (341 with UM only and 242 with CPM)	8
Pesce (2014)	Retrospective cohort	USA	All aged ≤45 years TNM I/II (100%) Grade 1 / 2 (50%; majority) HR+ (Estrogen receptor +) (70%) Median follow up of 6.1 years	14,627 (10289 with UM only and 4338 with CPM)	7
Jatoi (2014)	Retrospective cohort	USA	Mean age of around 60 years TNM I/II (89%) Grade 1 /2 (60%) HR+ (Majority; >60%) Mean follow up of around 5 years	449178 (423,217 with UM only and 25,961 with CPM)	8
Metcalfe (2014)	Retrospective cohort	Canada	Mean age of around 42 years TNM I/II (100%) Mean follow up of around 13 years	390 (209 with UM only and 181 with CPM)	7
Evans (2013)	Prospective cohort	United Kingdom	Mean age of around 40 years TNM I/II (85%) Grade 3 (67%) HR+ (Majority; 50%) Median follow up of around 7 years	210 (105 with UM only and 105 with CPM)	8
Yao (2013)	Retrospective cohort	USA	Majority aged more than 40 years TNM I/II (80%) Grade 1 /2 (60%) HR- (Majority; 65%) Mean follow up of 5.3 years	219983 (204989 with UM only and 14994 with CPM)	8
Brewster (2012)	Retrospective cohort	USA	Majority aged 50 years and above (60%) TNM I/II (70%) HR+ (78%) HER 2- (75%) Median follow up of 4.5 years	3889 (3357 with UM only and 532 with CPM)	7
King (2011)	Retrospective cohort	USA	Median age of around 50 years TNM I/II (majority) HR+ (75%) HER 2- (80%) Median follow up of 4.4 years	2979 (2572 with UM only and 407 with CPM)	7
Kiely (2010)	Retrospective cohort	Australia	Mean age of around 47 years Stage not reported Grade not reported Hormonal receptor status not reported Mean follow up of 11 years	1018 (864 with UM only and 154 with CPM)	7
Bedrosian (2010)	Retrospective cohort	USA	Majority aged 50 years and above (70%) TNM I/II (Majority; 70%) Grade 1 / 2 (50%; majority) HR+ (57%) Median follow up of 47 months	107016 (98204 with UM only and 8902 with CPM)	8
Boughey (2010)	Retrospective cohort	USA	Majority aged 40 years and above (73%) TNM I/II (100%) HR+ (77%) Median follow up of 17.3 years	770 (385 with UM only and 385 with CPM)	8
van Sprundel (2005)	Retrospective cohort	Netherlands	Mean age of around 40 years TNM I/II (80%) Grade 3 (70%) HR- (75%) Mean follow up of 3.5 years	148 (69 with UM only and 79 with CPM)	8
Herrinton (2005)	Retrospective cohort	USA	Mean age of around 50 years Mean follow up of 5.7 years	1389 (317 with UM only and 1072 with CPM)	8

Eleven studies had reported on the grade of the tumour: seven had patient with grade three or four tumour and remaining four had patients with grade one or two tumour (Supplementary Table-I). Three studies had patients with triple negative tumour. Out of seven studies that reported HER-2 status, all were negative. There were 13 studies with most patients with hormonal receptor-positive (HR+) tumour and two studies had patients with HR- tumour. The follow up period varied among the studies and ranged from 35 months (roughly three years) to 17.3 years (Supplementary Table-I). NOS score of 13 studies was eight (out of a maximum attainable score of nine) and NOS of another eight studies was seven. The average NOS score of the included studies was 7.6, indicating that the studies were of acceptable quality.

### Overall survival (OS)

**Fig.2:**
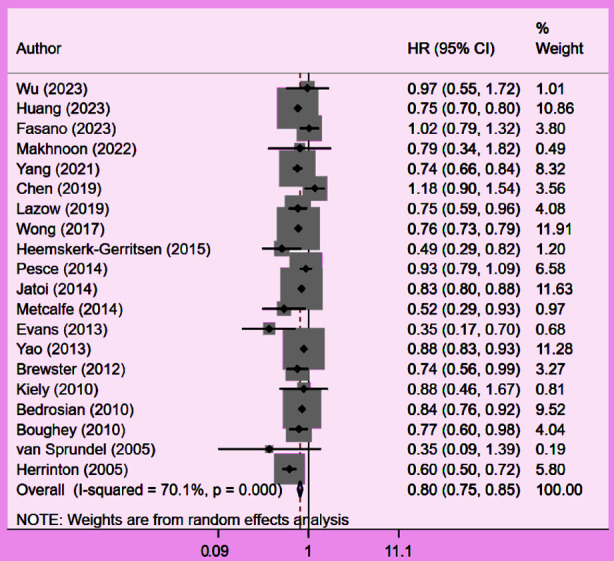
Association between contralateral prophylactic mastectomy and overall survival in subjects with unilateral breast cancer.

**Supplementary Fig.1:**
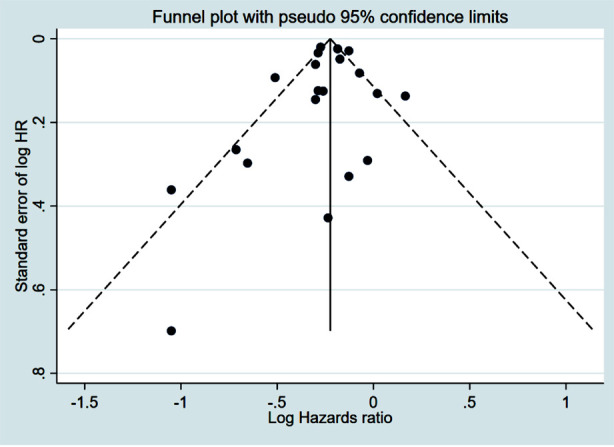
Funnel plot for publication bias assessment among studies examining the association between contralateral prophylactic mastectomy and overall survival in subjects with unilateral breast cancer.

Patients who underwent CPM had significantly improved OS (HR 0.80, 95% CI: 0.75, 0.85; N=20, I^2^=70.1%) (Fig.2), with no evidence of publication bias, as confirmed by funnel plots and the Egger’s test (p=0.58) (Supplementary Fig.1). The subgroup analysis showed that patients with early stage (Stage-I or II) tumour had better OS with CPM. CPM was associated with improved OS (Table-I), irrespective of the tumour grade. However, the association with improved OS was found only in HR+ patients, and not in HR- or triple negative patients (Table-I).

**Table-I:** Findings of the subgroup analysis.

	OS	BCSS	RFS	CBC
Stage I or II	0.81 (0.76, 0.86) * N=17; I^2^=70.2%	0.84 (0.77, 0.92) * N=11; I^2^=82.5%	0.72 (0.60, 0.86) * N=4; I^2^=45.1%	0.06 (0.03, 0.12) * N=6; I^2^=4.3%
Grade 1 or 2	0.85 (0.82, 0.89) * N=4; I^2^=17.2%	0.84 (0.79, 0.88) * N=2; I^2^=0.0%	----	----
Grade 3 or 4	0.75 (0.64, 0.87) * N=7; I^2^=58.8%	0.82 (0.65, 1.03) N=5; I^2^=91.4%	0.93 (0.69, 1.25) N=1	0.10 (0.04, 0.23) * N=3; I^2^=0.0%
Hormone receptor positive	0.81 (0.76, 0.87) * N=13; I^2^=73.2%	0.81 (0.75, 0.88) * N=7; I^2^=69.6%	0.67 (0.58, 0.78) * N=3; I^2^=0.0%	0.09 (0.04, 0.18) * N=4; I^2^=0.0%
Hormone receptor negative	0.84 (0.65, 1.08) N=4; I^2^=55.8%	0.94 (0.71, 1.24) N=3; I^2^=86.5%	0.93 (0.69, 1.25) N=1	0.10 (0.01, 0.88) * N=1
Triple negative breast cancer	0.86 (0.67, 1.11) N=3; I^2^=63.2%	0.80 (0.70, 0.91) * N=2; I^2^=0.0%	0.93 (0.69, 1.25) N=1	----

OS- Overall survival; BCSS-Breast cancer specific survival; RFS- recurrence free survival; CBC- contralateral breast cancer; *statistically significant at P<0.05.

### Breast cancer specific survival (BCSS)

**Fig.3:**
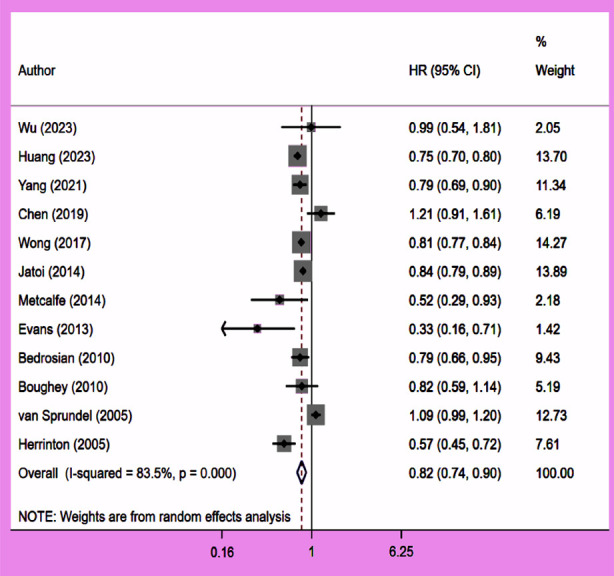
Association between contralateral prophylactic mastectomy and breast cancer specific survival in subjects with unilateral breast cancer.

**Supplementary Fig.2:**
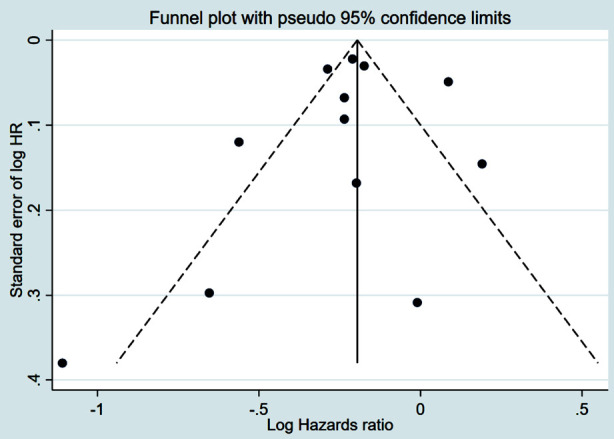
Funnel plot for publication bias assessment among studies examining the association between contralateral prophylactic mastectomy and breast cancer specific survival in subjects with unilateral breast cancer.

CPM correlated with improved BCCS (HR 0.82, 95% CI: 0.74, 0.90; N=12, I^2^=83.5%) (Fig.3), compared to patients who did not undergo the surgery, with no evidence of publication bias on Egger’s test (p=0.77) (Supplementary Fig.2). The subgroup analysis showed that patients with early-stage (Stage-I or II) and low-grade (Grade-1 or 2) tumours exhibited improved BCSS with CPM. Additionally, CPM was associated with better BCSS in HR+ patients, and triple negative patients (Table-I). However, this association with improved BCSS was not observed in patients with high-grade (Grade-2 or 3) tumours or HR-negative tumours (Table-I). For some of the performed analyses, the number of studies were few and therefore, statistical significance may not have been achieved, even if there was one.

### Recurrence free survival (RFS)

**Fig.4:**
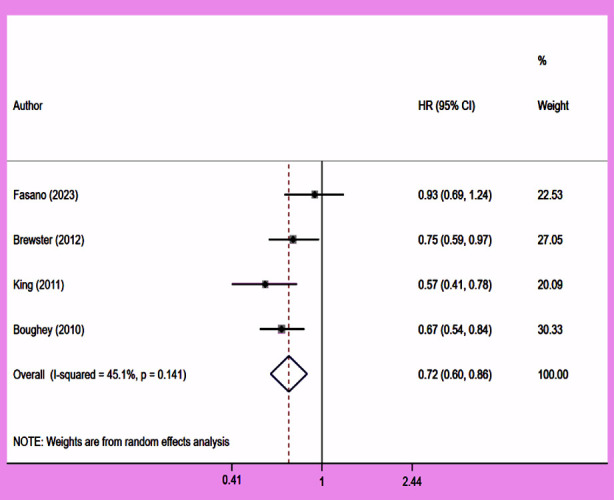
Association between contralateral prophylactic mastectomy and recurrence free survival in subjects with unilateral breast cancer.

**Supplementary Fig.3:**
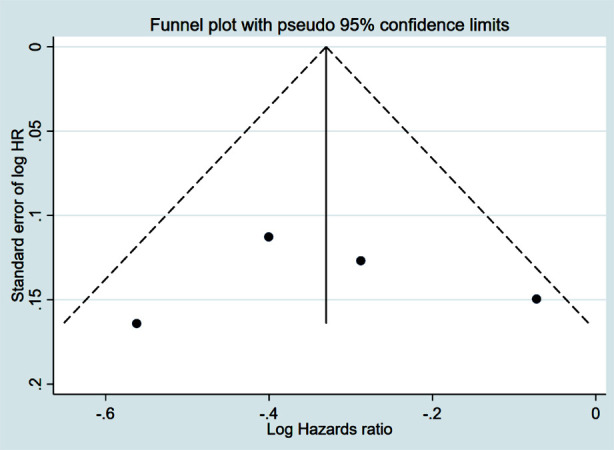
Funnel plot for publication bias assessment among studies examining the association between contralateral prophylactic mastectomy and recurrence free survival in subjects with unilateral breast cancer.

Compared to patients who were not operated on, CPM was associated with improved RFS (HR 0.72, 95% CI: 0.60, 0.86; N=4, I^2^=45.1%) (Fig.4), with no evidence of publication bias [Egger’s test (p=0.97)] (Supplementary Fig.3). The subgroup analysis revealed that patients with early-stage (Stage-I or II) tumour and HR+ tumour status had improved RFS with CPM (Table-I).

### Contralateral breast cancer (CBC)

**Fig.5:**
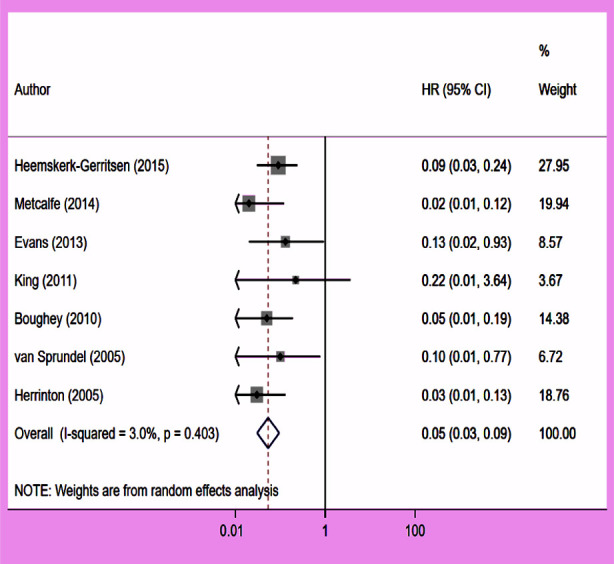
Association between contralateral prophylactic mastectomy and risk of contralateral breast cancer in subjects with unilateral breast cancer.

**Supplementary Fig.4:**
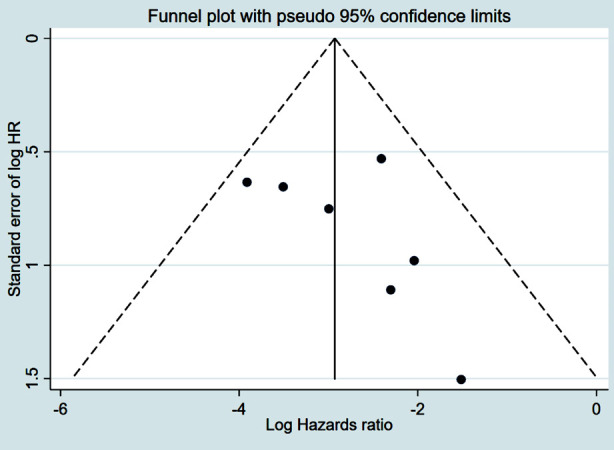
Funnel plot for publication bias assessment among studies examining the association between contralateral prophylactic mastectomy and risk of contralateral breast cancer in subjects with unilateral breast cancer.

CPM was associated with a significantly reduced risk of CBC (HR 0.05, 95% CI: 0.03, 0.09; N=7, I^2^=3.0%) (Fig.5). There was no evidence of publication bias, either on egger’s test (p=0.32) or on inspection of funnel plot (Supplementary Fig.4). The subgroup analysis revealed that this association was present in all subgroups, for which data were available from individual studies (Table-I).

## DISCUSSION

The findings of our meta-analysis provide compelling evidence supporting the favourable outcomes associated with CPM among women diagnosed with UBC. CPM was associated with significantly improved OS, BCSS, RFS, and lower risk of CBC. Our findings are similar to the previous two reviews on this issue.^7^,^8^ A review by Jia et al. revealed that CPM correlated with a decreased risk of CBC, underscoring the preventive nature of this surgical intervention. Moreover, the review highlighted a significant increase in both OS and BCSS among patients opting for CPM compared to those receiving no intervention on the contralateral breast. A study by Fayanju et al that included 14 studies showed a significantly higher OS in CPM patients compared to patients who did not undergo the surgery.^8^ Moreover, CPM was associated with lower rates of BCSS and CBC. CPM involves the surgical removal of the apparently healthy contralateral breast. Eliminating a potential site for cancer development appears to result in a substantial decrease in the probability of developing a new primary cancer in the contralateral breast.

This risk reduction may contribute to an improved OS rate. The decision to undergo CPM is often influenced by psychological factors, including anxiety and fear of cancer recurrence.^33^,^34^ For some women, the removal of the contralateral breast might alleviate psychological distress associated with the constant fear of developing a new cancer in the unaffected breast.^34^ The resulting reduction in psychological burden may positively impact overall well-being and contribute to a better quality of life, indirectly influencing OS, and contributing to an improved BCSS. UBC patients who opt for CPM possibly reduce the burden of dealing with bilateral disease. Bilateral involvement can complicate treatment approaches and present additional challenges, associated with managing two separate breast cancers. In such cases, opting for CPM may potentially lead to improved disease-specific outcomes.

There is limited data on the practice of conducting CPM. Available data is mostly from developed settings. A recent systematic review^4^ documented that in the USA, between 2004 and 2012, there was a nearly three-fold rise in CPM rates across all age groups, with the most substantial increase observed among women under 40 years old. CPM rates exhibited an inverse correlation with age, ranging from 3% in patients aged 70 or older to 30% in those aged 20 to 29. Factors significantly associated with opting for CPM included tumor type, particularly lobular histology, hormone receptor-positive (ER+/PR+) cancer, Caucasian ethnicity, and possessing private insurance. The primary motivations for undergoing CPM were to mitigate mortality risk and eliminate the potential of contralateral breast cancer occurrence. Increased societal attention toward breast cancer prevention, screening, and genetic testing may contribute to an overestimation of contralateral breast cancer risk among patients. Moreover, the review highlights the influential role of surgeons’ opinions in patients’ decisions regarding CPM.

In addition to exploring the impact of contralateral prophylactic mastectomy (CPM) on survival outcomes, a retrospective study by Fei et al.^35^, investigated the influence of a single esketamine intravenous (IV) injection on the recovery of breast cancer patients following modified radical mastectomy. The findings revealed that esketamine significantly improved the quality of early recovery. This suggests that esketamine, known for its anesthetic and analgesic properties, could be a beneficial adjunct in perioperative care, enhancing the immediate postoperative experience of mastectomy patients. While our primary focus remains on CPM’s impact on survival, acknowledging such studies is essential, as they contribute valuable insights into optimizing the holistic care of breast cancer patients during the immediate postoperative period.

### Limitations

Our analysis has some limitation which should be considered while interpreting the findings. The included studies exhibited considerable variations in sample sizes, population characteristics, tumour features, and follow-up durations, potentially introducing heterogeneity. Most of the studies in our review were retrospective. Therefore, there is a risk that essential confounders were not systematically collected or adjusted for. This may have introduced a potential bias in the observed associations.

While we specifically included studies reporting adjusted effect sizes, there were discrepancies in the variables adjusted for among the different studies, introducing some heterogeneity in the reported strength of associations. Additionally, most of the studies were conducted in limited geographical settings, potentially constraining the external generalizability and broader applicability of the findings. Finally, individual studies also differed in their surgical approaches to mastectomy, potentially influencing the outcomes.

## CONCLUSION

Our meta-analysis provides evidence supporting the positive impact of CPM on overall survival, BCSS, RFS, and the risk of CBC in women with UBC. These findings contribute valuable insights to the ongoing discourse on personalized treatment strategies and shared decision-making in the management of this malignancy. While the observed survival benefits with CPM are noteworthy, it is essential to consider the potential trade-offs, including surgical risks, cosmetic outcomes, and the impact on the quality of life. The decision to undergo CPM should be made through collaboration between patients and healthcare providers. This approach would allow for individualized treatment decisions based on factors such as patient preferences, risk tolerance, and perceived benefits. Further research and long-term follow-up studies are needed to validate our findings and refine recommendations for the use of CPM in specific clinical scenarios.

### Authors’ contributions:

**MY:** Conceived and designed the study.

**MY**, **PP**, **LC** and **ZX:** Collected the data and performed the analysis.

**MY:** Was involved in the writing of the manuscript and is responsible for the integrity of the study.

All authors have read and approved the final manuscript.
